# Effects of *Echinacea purpurea* and Alkylamides on Respiratory Virus Replication and IL-8 Expression In Vitro

**DOI:** 10.3390/molecules30020386

**Published:** 2025-01-17

**Authors:** Keely Puchalski, Johanne A. Gerstel, Abiola Jimoh, Yalda Shokoohinia, Jeffrey Langland

**Affiliations:** Ric Scalzo Institute for Botanical Research, Sonoran University of Health Sciences, Tempe, AZ 85282, USA; k.puchalski@sonoran.edu (K.P.); j.gerstel@sonoran.edu (J.A.G.); aajimoh24@gmail.com (A.J.); yalda.shokooh@nowfoods.com (Y.S.)

**Keywords:** *Echinacea*, alkylamides, rhinovirus, influenza, immunology, interleukin-8, virology

## Abstract

*Echinacea purpurea* is a perennial medicinal herb with important immunomodulatory and anti-inflammatory properties, especially purported for the alleviation of cold and flu symptoms. Different classes of secondary metabolites of the plant, such as alkylamides, caffeic acid derivatives, polysaccharides, flavonoids, and glycoproteins, are believed to be biologically and pharmacologically active. Although previous research suggests that the alkylamides present in *Echinacea* may be responsible for reducing the symptoms associated with the common cold or flu through their immunomodulatory activity, the roles of specific alkylamides and their targets (i.e., immune and/or antiviral) have not been well-elucidated or established. This study tested the antiviral and cytokine regulatory activity of various specific alkylamides that are present predominantly in *Echinacea* root extracts and found that one specific alkylamide, Dodeca-2*E*,4*E*-Dienoic acid isobutylamide, had potent antiviral activity against rhinovirus (the causative agent of most common colds) and influenza virus, as well as potent inhibition of IL-8 cytokine production. IL-8 is responsible for many of the symptoms associated with the common cold and is upregulated in other common respiratory infections. The broad activity and low cytotoxicity of this specific alkylamide support its potential use for treating rhinovirus and influenza virus infections.

## 1. Introduction

*Echinacea* is one of the most popular Western herbs used in both traditional and modern medicine. A member of the *Asteraceae* family native to North America. *Echinacea*’s first documented uses were by several Great Plains tribes, including the Lakota, Cheyenne, and Sioux, among others [1,2,3,4,5]. These tribes used the plant to treat a wide variety of ailments, including respiratory tract infections (RTIs), wounds, toothaches, and snake bites [1,3]. In the late 1800s, a German American doctor known as H.C.F. Meyer marketed a commercial tincture of *Echinacea,* known as “Meyer’s Blood Purifier”, as a cure-all based on indigenous knowledge, and in the early 1900s, a group of physicians known as the Eclectics began to use *Echinacea* more broadly and published several articles on its benefits [3]. Though the favored remedy took a backseat to antibiotics and other pharmaceuticals in the 1940s and was removed from the U.S. Pharmacopeia (USP) in 1945, it saw a significant resurgence in popularity in the 1970s and 1980s [3]. Its use spread further during the 1990s when a flood of *Echinacea* supplements hit the market and scientists began conducting more research on its potential benefits [6,7,8]. *Echinacea* has remained in the limelight into the 21st century thanks to continued research efforts and consistent consumer demand. According to market reports, *Echinacea* continues to rank among the top 10–20% of all U.S. herbal supplements in terms of sales, demonstrating its long-established position in Western herbal medicine [8,9,10,11].

Though *Echinacea* was historically touted as a “cure all”, it is most widely utilized and researched today for immune system modulation and the prevention of and recovery from respiratory infections like the common cold and flu. According to the Centers for Disease Control and Prevention (CDC), adults in the U.S. suffer from common colds an average of 2–3 times per year and children as frequently as 6–8 times, to the sum of over 1 billion colds each year [12,13,14]. The flu is less common, infecting adults once every 3–5 years, on average, and children and the elderly slightly more frequently [15,16]. Though less common, the flu poses a greater risk for complications in at-risk populations, which include the elderly, immunocompromised individuals, young children, and pregnant women. Complications of the flu include asthma exacerbations and secondary infections like otitis media, sinus infections, and pneumonia, the most serious of which can lead to hospitalizations and in severe cases, death [15,17,18]. Colds generally produce milder symptoms and are less fatal. However, they can also lead to secondary infections and more severe symptoms in those with comorbidities [12]. RTIs, regardless of severity, cause significant disruptions to work and school activities, costing the U.S. economy billions of dollars each year [14,16,19,20].

The common cold is caused by over 200 different viruses, most of which are members of the rhinovirus family [12,21,22]. When the rhinovirus infects epithelial cells in the nasal passages of the upper respiratory tract, one of the predominant cellular responses is the release of a cytokine called interleukin-8 (IL-8) [23,24]. IL-8 is a signaling protein that recruits neutrophils, a type of white blood cell, to the infection site, which induces an inflammatory response. The IL-8-induced inflammatory response and tissue changes, rather than cell damage from the virus itself, are thought to cause cold symptoms, which generally include rhinorrhea or congestion, sore throat, sneezing, and a cough [12,23,24,25]. Several other cytokines have been shown to be involved in rhinovirus infections, including interferons, IL-1, IL-6, and TNF-α, but IL-8 has consistently been demonstrated to be the key player associated with producing the symptoms of the common cold and is involved to some degree in the immune response to other common RTIs as well [24,26,27]. Common colds usually last around 7–10 days, and do not respond to antibiotic treatment, as they are viral infections [12,16]. Antibiotics are sometimes prescribed for RTIs, particularly if the infectious etiology is unclear (i.e., viral rhinosinusitis vs. bacterial sinusitis), or as palliative care. Identifying novel treatments for rhinovirus infections that inhibit viral replication and lessen symptom severity and duration is important for improving patient quality of life and reducing economic losses.

Unlike the common cold, which is caused by many viruses, the flu is caused by evolving strains of influenza viruses that develop due to genetic changes in the virus’s antigens [28]. These genetic changes include minor antigenic drifts or, rarely, major antigenic shifts. Antigenic shifts occur when flu genes from different species infect a single host and interact to create entirely new viral subtypes. These “novel” strains or subtypes can result in more serious infections and flu pandemics, as the population has usually never been exposed to them and has little or no prior immunity [29]. Flu symptoms have an abrupt onset and commonly include early systemic symptoms of fatigue, cough, myalgias, and sometimes fever or headache, followed by later respiratory symptoms including shortness of breath, wheezing, or chest pain [15,17,30,31]. Both rhinovirus and influenza virus infections tend to peak within 2 days, earlier than most other respiratory viruses [31]. Seasonal vaccines are recommended as the gold standard for flu prevention but vary in efficacy depending on the strain and timing of administration [28,29,32]. Treatments for the flu are also limited, as they must be given early in the course of illness (within 48 h of symptom onset), can be difficult to obtain, may lead to antiviral resistance, and have not been shown to significantly impact outcomes for otherwise healthy populations [33,34,35,36]. Identifying accessible and effective treatments for the flu, much like treatments for rhinovirus, is necessary for improving patient quality of life and reducing disruptions to daily activities. Furthermore, dependable flu treatments are critical for preventing complications and mortality in at-risk populations and during novel flu strain pandemics.

The collective clinical and pre-clinical evidence for *Echinacea*’s use in RTI prevention and treatment is mixed and, therefore, controversial. Although there are a few dozen clinical trials published on *Echinacea* and RTIs, the products and preparations in these studies vary considerably, and firm conclusions remain elusive. Many of these studies exhibit small sample sizes and study design flaws or report inconclusive or nonsignificant results. Several systematic reviews and meta-analyses have been conducted on *Echinacea* in the past decade, including the most recently updated 2014 Cochrane review on *Echinacea* for the prevention and treatment of the common cold [37]. The Cochrane review concluded that there was no plausible evidence for the treatment of the common cold. However, “at least some *Echinacea* preparations may reduce the relative risk of catching a cold by 10% to 20%”. Notably, the 24 studies included in that review employed an appreciable variety of products, primarily alcohol tinctures, tablets from dried extracts, and pressed juices. Products were derived from either the root, aerial parts, or both, and only some were standardized to one or more different constituents in varying percentages. This heterogeneity, along with a significant number of nondisclosed preparation methods, manufacturers, and extraction methods, was cited as the primary reason for the lack of conclusive evidence regarding treatment efficacy (i.e., reduction of cold duration and severity) [37]. Several additional reviews have reported similar findings to the Cochrane review, noting *Echinacea*’s possible role in the prevention, but not treatment, of RTIs, and reiterating the need for larger trials with stronger methods [38,39,40,41,42,43]. A 2024 meta-analysis by Gancitano et al. investigated 30 clinical trials involving 5652 subjects and concluded that *Echinacea* was effective in preventing RTIs, as well as reducing the secondary complications of RTIs, leading to an overall reduction in the need for antibiotic therapy (70% reduction in total antibiotic therapy days) [39]. A smaller 2015 meta-analysis of six studies by Schapowal et al. also reported successful prevention of RTIs and a reduction in RTI complications, particularly in those with confirmed viral infections and those with increased susceptibility to recurrent RTIs [41]. In terms of *Echinacea*’s role in cytokine regulation during RTIs, a systematic review of 105 studies (13 human, 24 animal, and 71 in vitro or ex vivo) by Aucoin et al. concluded that “*Echinacea* supplementation may be associated with a decrease in the pro-inflammatory cytokines IL-6, IL-8, and TNF-α, as well as an increase in the anti-inflammatory cytokine IL-10”. However, a high risk of bias overall was mentioned [40]. To summarize, most clinical reviews point towards *Echinacea*’s potential role in RTI prevention and report that the herb is likely safe for most populations when used in the short term [38,41,44,45,46,47,48]. In terms of treatment for RTIs, more studies are needed to clarify which plant parts, extraction methods, and specific compounds could be responsible for the positive outcomes seen in the mixed clinical results to date

Most *Echinacea* products on the market today contain *E. purpurea*, *E. pallida* var. *angustifolia* (syn. *E. angustifolia*), *E. pallida* var. *pallida* (syn. *E. pallida*), or a combination of these species [47,49,50]. Most of the literature to date has investigated *E. purpurea* products made from the roots and/or aerial parts, followed by studies on *E. angustifolia* [8,47,48]. Several secondary metabolites from *Echinacea* have been investigated for potential pharmacological activity, including polysaccharides, glycoproteins, caffeic acid derivatives, flavonoids, and alkylamides [50,51]. The amount of these constituents varies by plant part and species [6,52,53]. A 2022 review on the biological and pharmacological properties of *E. purpurea* by Burlou-Nagy et al. included a comprehensive summary of each chemical group’s purported activities, including up to ten different actions reported within each class [6]. Though it remains unclear which compounds are directly or indirectly involved in certain biological effects, the in vitro data in particular demonstrates that *E. purpurea* compounds and extracts possess potent antiviral, anti-inflammatory, and immunomodulatory activities [6,47,51,54,55,56,57,58,59].

Of the potential therapeutic compounds, the caffeic acid derivatives (CADs) and alkylamides have been most studied and are both routinely used as marker compounds for authenticating *Echinacea* plant material and in quality control practices [51,60,61,62]. CADs, like other polyphenols, naturally act as strong antioxidants but have also been shown to have a broad range of other activities, particularly the CADs echinacoside, cichoric acid, and caftaric acid, which are thought to contain immunomodulatory and antiviral activity based on in vitro studies [58,63,64,65]. Cichoric acid is the most prevalent compound in *Echinacea* plants, and echinacoside and cichoric acid are most commonly seen and identified in commercial dietary supplements [58].

The lipophilic alkylamides have gained increasing attention in recent decades, largely because they are the only compounds in *Echinacea* known to pass through the intestinal tract into the bloodstream and they have been shown to possess cannabinomimetic activity via CB2 receptor agonism, which demonstrated a new possible mechanism for their purported immunomodulatory activity [54,55,66,67,68,69,70,71]. In vitro studies have also demonstrated alkylamide-induced modulation of monocytes and macrophages, upregulation of NF-κB, induction of IL-10, reduction of NO, and inhibition of IL-2, lipopolysaccharide-induced TNF-α, COX-1, and to a lesser extent, COX-2, suggesting that these compounds possess significant immunomodulatory and anti-inflammatory properties [48,51,55,57,69,72,73,74]. A handful of in vivo mouse and rat studies and ex vivo human studies after in vivo oral administration have supported many of the above findings [49]. Beyond immune modulation and anti-inflammatory activity, a recent review of *Echinacea*’s biological activities reported that alkylamides found in *Echinacea* have also demonstrated antiviral, antimicrobial, antioxidant, and antiosteporotic activity, though direct mechanisms for these are unclear [6].

Alkylamides are most prevalent in the root bark and secondary roots of *E. purpurea* and *E. angustifolia*, at concentrations of up to 6 mg/g in the highest quality plant material, followed by lesser amounts in the aerial parts of *E. purpurea*, *E. angustifolia*, and *E. pallida* [49,75,76,77]. Bauer and Remiger (1989) originally identified 25 alkylamides in *E. purpurea* and *E. angustifolia* via TLC and HPLC and numbered them 1 through 25 [78]. This numeric system is often still utilized today, especially numbers 8 and 9, which represent the two most prevalent alkylamides isolated from *E. purpurea* (Dodeca-2*E*,4*E*,8*Z*,10*E*(*Z*)-tetraenoic acid isobutylamides) [78,79]. At least 11 of the 25 total alkylamides identified by Bauer and Remiger (1989) were found in *E. purpurea* roots and 16 of them in *E. angustifolia* roots. A later 2009 publication by Spelman et al. comparing alkylamide yield in ethanolic *E. purpurea* extracts from fresh versus dry plant material utilizing HPLC-ESI-MS included a table with 17 different alkylamides previously discovered in *E. purpurea* [80]. The most recent alkylamide overview by Mudge et al. (2011) utilized UFLC-DAD-MS analysis and identified 24 total alkylamides in the roots of *E. purpurea* and *E. angustifolia*, including 15 of the 17 previously reported in *E. purpurea* by Spelman et al. (2009), and 22 in *E. angustifolia* [77]. Of the identified alkylamides in *E. purpurea* to date, only a few have been investigated for pharmacological activity, primarily numbers 8 and 9 (Dodeca-2E,4E,8Z,10E(Z)-tetraenoic acid isobutylamides), as well as three other alkylamides that are found in higher concentrations in *Echinacea* roots: undeca-2E-ENE-8,10-diynoic acid isobutylamide, dodeca-2E-ENE-8,10-diynoic acid isobutylamide, and dodeca-2E,4*E*-dienoic acid isobutylamide [48,55,56,63,66,69]. For simplicity, in this manuscript, these alkylamides are labeled as A (8/9 isomers), B, C, and D, respectively.

Our research investigated the ability of ethanolic extracts from *E. purpurea* roots and various individual alkylamides to inhibit rhinovirus and influenza virus replication, as well as modulate IL-8 expression in vitro. We discovered that alkylamide D, dodeca-2*E*, and 4*E*-dienoic acid isobutylamide displayed potent inhibition of both viruses, as well as IL-8 expression, suggesting this alkylamide could be further investigated as a potential therapeutic for both the common cold and flu. To our knowledge, none of the alkylamides identified in *Echinacea* have been investigated for direct inhibition of antiviral activity against rhinovirus or influenza virus, and only three of the four tested alkylamides in this study (A, B, and D) were previously investigated for IL-8 modulation [69].

## 2. Results

### 2.1. Effect of E. purpurea Extract on Rhinovirus and Influenza Virus Replication

**Figure 1:**
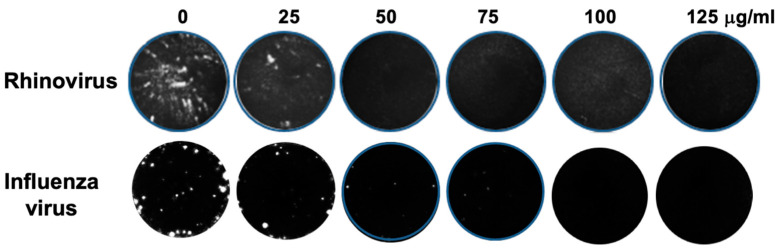
Viral plaque assay showing inhibitory effect of *Echinacea purpurea* extract on rhinovirus and influenza virus replication. HeLaH1 or MDCK cells were infected with either rhinovirus or influenza virus, respectively, in the presence of increasing concentrations (0–125 μg/mL) of *Echinacea purpurea* 70% ethanol root extract. After 72 h of infection, cells were fixed and stained with crystal violet to visualize viral plaques. Representative images of plaques from each treatment group are shown. Viral plaques were counted and normalized to untreated controls (0 µg). Vehicle controls (70% ethanol) were tested at the same volumes, corresponding to the treatment doses, and the results for the maximum volume used are graphed in Figure 2. Results did not differ from untreated controls and had no effect on the treatment outcomes.

To examine the effects of an *E. purpurea* root extract on rhinovirus and influenza virus replication, viral plaque assays were performed. HeLaH1 or MCDK cells were infected with either rhinovirus or influenza virus, respectively, in the presence of increasing concentrations of the *Echinacea* extract (0–125 μg/mL). As shown in Figure 1, the *Echinacea* extract reduced both rhinovirus and influenza virus replication compared to untreated controls, as indicated by the reduction and absence of viral plaque formation with increasing treatment concentrations. Plaques were counted and graphed as a reduction in the percentage of viral plaque formation (See subsequent figures). A significant reduction in plaque formation was observed at 25 μg/mL (*p* < 0.001 for rhinovirus and *p* < 0.01 for influenza virus) and 50 μg/mL (*p* < 0.001 for both viruses). Complete viral inhibition was observed at 75 μg/mL for both viruses.

### 2.2. Effect of Phenolics from E. purpurea on Rhinovirus Replication

**Figure 2:**
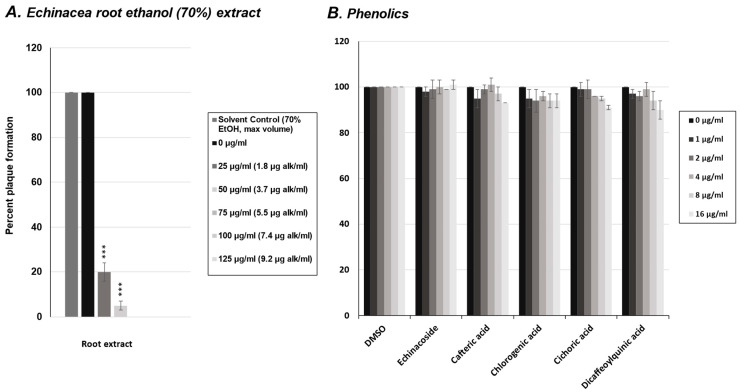
Rhinovirus plaque reduction assay with *Echinacea* extract and phenolics. (**A**) Quantification of plaque numbers from rhinovirus plaque assay treated with *Echinacea* crude extract from Figure 1. (**B**) HeLaH1 cells were infected with rhinovirus in the presence of increasing concentrations (0–16 μg/mL) of five phenolic compounds: echinacoside, cafteric acid, chlorogenic acid, cichoric acid, and dicaffeoylquinic acid. After 72 h of infection, cells were fixed and stained with crystal violet to visualize plaques. Viral plaques were counted and normalized to untreated controls (0 µg). Statistical significance was determined using a one-sample *t*-test. Significant differences are indicated by *p* < 0.001 (***). Error bars represent the mean ± SD (*n* = 3). Vehicle controls (70% ethanol for the crude extract) were tested at the same volumes, and the results for the maximum volume used are shown in (**A**). For phenolic compounds (**B**), DMSO vehicle doses corresponding to the tested concentrations are shown to account for any potential dose-dependent effects of the solvent. These controls did not differ from untreated controls and had no effect on the treatment outcomes.

To investigate potential antiviral constituents in *E. purpurea* root extracts against rhinovirus, we first tested five of the most common phenolic compounds in a viral plaque assay. The phenolics tested included echinacoside, cafteric acid, chlorogenic acid, cichoric acid, and dicaffeoylquinic acid. HeLaH1 cells were infected with rhinovirus in the presence of increasing concentrations of each phenolic compound (0–16 μg/mL). Viral plaques were counted, and as shown in Figure 2B, none of the phenolics tested significantly reduced rhinovirus plaque formation compared to the untreated controls, nor compared to the plaque reductions observed with the crude *E. purpurea extract* (Figure 2A).

### 2.3. Effect of Alkylamides from E. purpurea on Rhinovirus Replication

**Figure 3:**
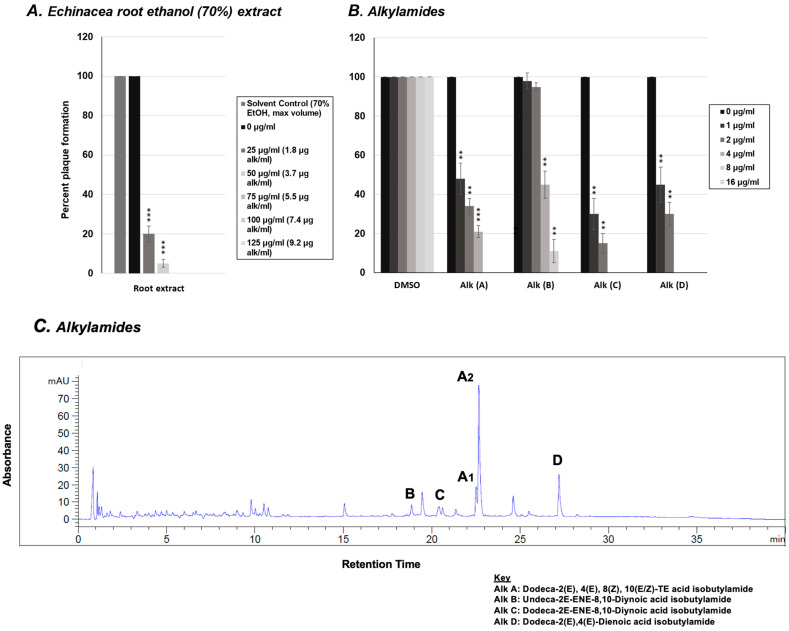
Rhinovirus plaque reduction assay with *Echinacea* extract and alkylamides. (**A**) Quantification of plaque numbers from rhinovirus plaque assay treated with *Echinacea* crude extract from Figure 1. (**B**) HeLaH1 cells were infected with rhinovirus in the presence of increasing concentrations (0–16 μg/mL) of various alkylamide compounds: A, B, C, and D (see key for compound names). After 72 h of infection, cells were fixed and stained with crystal violet to visualize plaques. Viral plaques were counted and normalized to untreated controls (0 µg). Statistical significance was determined using a one-sample *t*-test. Significant differences are indicated by *p* < 0.01 (**), and *p* < 0.001 (***). Error bars represent the mean ± SD (*n* = 3). (**C**) LC-MS analysis of the crude ethanolic extract was performed using UV detection at 330 nm, with the four alkylamide peaks (A–D) measured as absorbance values in milli-absorbance units (mAU). Alkylamides A1 and A2 represent *E*/*Z* isomers of alkylamide A (Dodeca-2*E*,4*E*,8*Z*,10(*E*/*Z*)-TE acid isobutylamide). When the activity of the alkylamides (**B**) was compared to the concentrations of alkylamides quantified in the crude *Echinacea* extract ((**A**), alk/mL), comparable concentrations of total alkylamides present in the crude extract had similar levels of activity relative to the pure alkylamides (with a viral IC80 around 2 µg/mL for both the crude extract and alkylamides C and D). Vehicle controls (70% ethanol for the crude extract) were tested at the same volumes, and the results for the maximum volume used are shown in (**A**). For alkylamide compounds (**B**), DMSO vehicle doses corresponding to the tested concentrations are shown to account for any potential dose-dependent effects of the solvent. These controls did not differ from untreated controls and had no effect on the treatment outcomes.

In addition to the five phenolic compounds, we tested the effects of four common alkylamides found in *E. purpurea* roots on rhinovirus replication using a viral plaque assay. HeLaH1 cells were infected with rhinovirus in the presence of increasing concentrations of each alkylamide compound (0–16 μg/mL). The viral plaques were counted, and as shown in Figure 3B, all four alkylamides demonstrated some antiviral activity with alkylamides C and D (Dodeca-2*E*-ENE-8,10-Diynoic acid isobutylamide and Dodeca-2*E*,4*E*-Dienoic acid isobutylamide, respectively) demonstrating the most significant activity (*p* < 0.01 at both 1 and 2 μg/mL doses). Identification and concentration of total alkylamides present in the ethanolic extract were obtained via LC-MS (Figure 3C). When the activity of the alkylamides (Figure 3B) was compared to the concentrations of alkylamides quantified in the crude *Echinacea* extract (Figure 3A: alk/mL), comparable concentrations of total alkylamides present in the crude extract had similar levels of activity relative to the pure alkylamides (with a viral IC_80_ around 2 μg/mL for both the crude extract and alkylamides C and D). This may suggest that the alkylamides present in the crude *Echinacea* extract may be responsible for the anti-rhinovirus activity, but the level of antiviral activity does vary amongst the different alkylamides.

### 2.4. Effect of Phenolics from E. purpurea on Influenza Virus Replication

**Figure 4:**
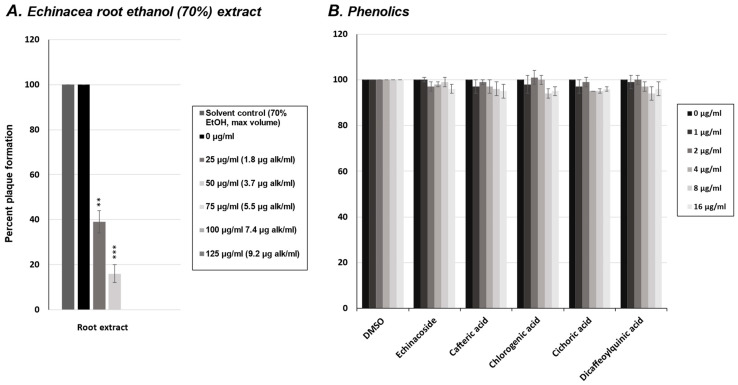
Influenza plaque reduction assay with *Echinacea* extract and phenolics. (**A**) Quantification of plaque numbers from influenza virus plaque assay treated with *Echinacea* crude extract in Figure 1. (**B**) MDCK cells were infected with influenza virus in the presence of increasing concentrations (0–16 µg/mL) of various phenolic compounds: echinacoside, cafteric acid, chlorogenic acid, cichoric acid, and dicaffeoylquinic acid. After 72 h of infection, cells were fixed and stained with crystal violet to visualize plaques. Viral plaques were counted and normalized to untreated controls (0 µg). Statistical significance was determined using a one-sample *t*-test. Significant differences are indicated by *p* < 0.01 (**), and *p* < 0.001 (***). Error bars represent the mean ± SD (*n* = 3). Vehicle controls (70% ethanol for the crude extract) were tested at the same volumes, and the results for the maximum volume used are shown in (**A**). For phenolic compounds (**B**), DMSO vehicle doses corresponding to the tested concentrations are shown to account for any potential dose-dependent effects of the solvent. These controls did not differ from untreated controls and had no effect on the treatment outcomes.

To investigate the potential antiviral constituents in *E. purpurea* root extracts against the influenza virus, we again tested five of the most common phenolic compounds in a viral plaque assay. The phenolics tested were the same as those tested against the rhinovirus (echinacoside, cafteric acid, chlorogenic acid, cichoric acid, and dicaffeoylquinic acid). MDCK cells were infected with the influenza virus in the presence of increasing concentrations of each phenolic compound (0–16 μg/mL). Viral plaques were counted, and as shown in Figure 4B, none of the phenolics tested significantly reduced influenza virus plaque formation compared to the untreated controls, nor compared to the plaque reductions observed with the crude *E. purpurea* extract (Figure 4A). Similar to our results in Figure 2, none of the phenolics were effective at inhibiting the rhinovirus or the influenza virus.

### 2.5. Effect of Alkylamides from E. purpurea on Influenza Virus Replication

**Figure 5:**
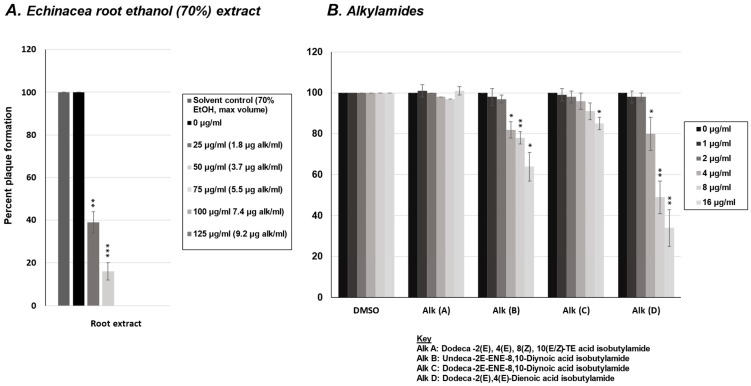
Influenza plaque reduction assay with *Echinacea* extract and alkylamides. (**A**) Quantification of plaque numbers from influenza virus plaque assay treated with *Echinacea* crude extract in Figure 1. (**B**): MDCK cells were infected with influenza virus in the presence of increasing concentrations (0–16 µg/mL) of various alkylamide compounds: A, B, C, and D (see Key for actual compound names). After 72 h of infection, cells were fixed and stained with crystal violet to visualize plaques. Viral plaques were counted and normalized to untreated controls (0 µg). Statistical significance was determined using a one-sample *t*-test. Significant differences are indicated by *p* < 0.05 (*), *p* < 0.01 (**), and *p* < 0.001 (***). Error bars represent the mean ± SD (*n* = 3). Vehicle controls (70% ethanol for the crude extract) were tested at the same volumes, and the results for the maximum volume used are shown in (**A**). For alkylamide compounds (**B**), DMSO vehicle doses corresponding to the tested concentrations are shown to account for any potential dose-dependent effects of the solvent. These controls did not differ from untreated controls and had no effect on the treatment outcomes.

In addition to the five phenolic compounds, we tested the effects of four common alkylamides found in *E. purpurea* roots on influenza virus replication using a viral plaque assay. MDCK cells were infected with influenza H1N1 in the presence of increasing concentrations of the four alkylamides. As shown in Figure 5B, alkylamide D (Dodeca-2*E*,4*E*-Dienoic acid isobutylamide) had the strongest antiviral activity against the influenza virus, with an IC_50_ of approximately 8 μg/mL (*p* < 0.01). The other alkylamides demonstrated little to no anti-influenza virus activity. When comparing the activity of alkylamide D (Figure 5B) to the activity of the crude *Echinacea* extract (Figure 5A), the total extract had a more potent anti-influenza virus activity relative to the total alkylamide concentration, having an IC_50_ of approximately 1.5 μg total alkylamides/mL. This suggests that either a different alkylamide or a different secondary metabolite may be responsible for some of the anti-influenza virus activity observed in the crude extract.

### 2.6. Regulation of LPS-Induced IL-8 Secretion with E. purpurea Extract and Alkylamides

**Figure 6:**
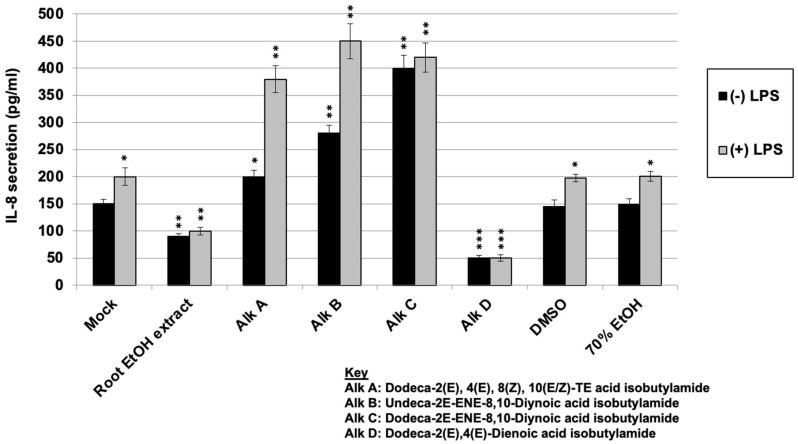
Regulation of LPS-induced IL-8 secretion with *Echinacea* extract and alkylamides. Interleukin-8 (IL-8) levels were measured by ELISA in human THP-1 cells treated with varying concentrations of *Echinacea* root 70% ethanol extract (50 µg/mL) or the various alkylamides (1 µg/mL) in the presence or absence of LPS (1 µg/mL). Statistical significance was determined using a one-sample *t*-test. Significant differences are indicated by *p* < 0.05 (*), *p* < 0.01 (**), and *p* < 0.001 (***). Error bars represent the mean ± SD (*n* = 3). Vehicle controls (70% ethanol and DMSO) were tested at the same volumes corresponding to the treatment doses, both with and without LPS, and were found to have no significant effect on IL-8 production compared to mock controls.

After investigating the direct antiviral activity and determining that the *E. purpurea* root 70% EtOH extract and alkylamides C and D demonstrated the ability to inhibit rhinovirus and the *E. purpurea* extract and alkylamide D inhibited/partially inhibited influenza virus replication, we decided to also investigate the potential immunomodulatory properties by evaluating IL-8 expression in human macrophages. ELISAs were conducted to investigate the effect of alkylamides compared to *E. purpurea* extract treatment on IL-8 synthesis in immune resting or stimulated cells. THP-1 cells were treated with the *Echinacea* extract or the various alkylamides followed by treatment or no treatment with an immune stimulant, lipopolysaccharide (LPS). The level of IL-8 production was measured by ELISA. LPS is a powerful activator of the immune response leading to the production of various cytokines, including IL-8. As shown in Figure 6, the treatment of cells with LPS alone (Mock + LPS) led to an induction in IL-8 secretion (compared to an untreated control (Mock) without LPS treatment) (*p* < 0.05). Treatment with *Echinacea* root ethanol extract led to a significant reduction in IL-8 levels in both the presence and absence of LPS treatment (*p* < 0.01 in both cases) compared to the untreated controls. This supports the previous results, where *Echinacea* extracts are thought to induce anti-inflammatory responses. Since alkylamides are thought to be responsible for the immune modulatory effects of *Echinacea*, the cells were treated with various purified alkylamides in the presence and absence of LPS. As shown in Figure 6, alkylamides A, B, and C (Dodeca-2*E*,4*E*,8*Z*,10(*E*/*Z*)-TE acid isobutylamide; Undeca-2*E*-ENE-8,10-Diynoic acid isobutylamide; and Dodeca-2*E*-ENE-8,10-Diynoic acid isobutylamide, respectively) all led to significant increases in IL-8 levels, both in the absence and presence of LPS, suggesting an immunostimulatory effect (*p* < 0.01–*p* < 0.05 depending on the treatment). When the cells were treated with alkylamide D (Dodeca-2*E*,4*E*-Dienoic acid isobutylamide), a significant reduction in IL-8 secretion was observed, both in the absence and presence of LPS (*p* < 0.001 for both). This effect was similar to that observed with the crude *Echinacea* extract but more significant. These results demonstrate that IL-8 expression modulation varies amongst the different alkylamides.

### 2.7. Selectivity Index Analysis of E. purpurea Extracts and Alkylamides

**Figure 7:**
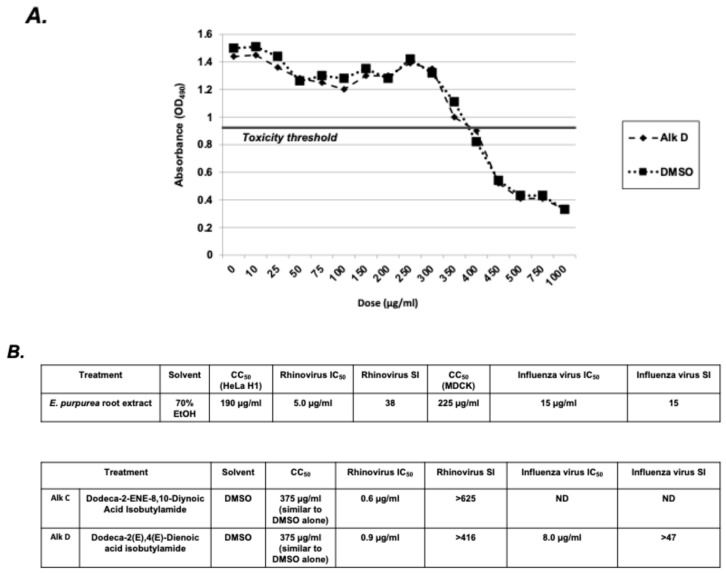
CC_50_ and IC_50_ of *E. purpurea* root extract and effective alkylamides. (**A**) To determine the CC_50_ of alkylamides C (Dodeca-2*E*-ENE-8,10-Diynoic acid isobutylamide) and D (Dodeca-2*E*,4*E*-Dienoic acid isobutylamide) and the *E. purpurea* EtOH extract, MTS assays were performed. HeLaH1 or MDCK cells were dosed with increasing concentrations (10–1000 µg/mL) of either *E. purpurea* extract or individual alkylamides and were incubated for 24 h, followed by addition of the MTS reagent. Absorbance was measured using a microplate reader. (**B**) The CC_50_ was determined by plotting the treatment concentration (x axis) vs. cell viability (y axis) dose–response curve and analyzing viability as a percentage of the control. The IC_50_ was determined from the viral plaque assay results in Figure 3 and Figure 5. The selectivity index (SI) was then calculated.

The antiviral activities and effects on IL-8 secretion of alkylamides A–D are compared in Table 1. Since alkylamide D (Dodeca-2*E*,4*E*-Dienoic acid isobutylamide) had significant antiviral activity against both the rhinovirus and the influenza virus and inhibited IL-8 secretion, this alkylamide has potential for use as a therapeutic for these infections. To initially assess if a compound has potential as a pharmacological agent, the selectivity index (SI) is often determined. The SI is a ratio between the cytotoxicity and antiviral activity of a potential drug. The higher the SI ratio, theoretically, the more effective and safer a therapeutic is during in vivo treatment for a given viral infection. Typically, a relatively low SI (<1) means that the compound could be toxic and should not be used as a therapeutic. If the calculated SI value is between 1 and 10, some concern is warranted, and the compound may be re-evaluated using other biosystems. Calculated SI values > 10 are desirable and have more potential therapeutic value. As shown in Figure 7, the SI for alkylamide D (Dodeca-2*E*,4*E*-Dienoic acid isobutylamide) is >47 for the influenza virus and >416 for the rhinovirus. These SI values are likely substantially higher since the CC_50_ for DMSO (the vehicle that the alkylamide is resuspended in) is the same as that for alkylamide D. This suggests that the observed cell cytotoxicity is likely due to the DMSO and not the alkylamide. Therefore, the SI for alkylamide D (Dodeca-2*E*,4*E*-Dienoic acid isobutylamide) is likely higher than reported. Of note, as shown in Figure 7, the SI for the *E. purpurea* root extract, which was also effective against both viruses and at reducing IL-8, was also very high. These SI values support the potential efficacy and safety of *E. purpurea* root extracts and alkylamide D (Dodeca-2*E*,4*E*-Dienoic acid isobutylamide), specifically, for the treatment of rhinovirus and influenza virus infections.

## 3. Discussion

Alkylamides from *Echinacea* have been shown to be the most bioavailable and pharmacologically active secondary metabolites present in the plant extracts [49,66,67,71]. Our research involved the precise testing of four individual alkylamides, providing valuable mechanistic insights into their antiviral and anti-inflammatory effects. By testing multiple compounds, these data offer novel comparisons of specific compound efficacy against the rhinovirus and the influenza virus and an ability to modulate IL-8 expression. Evaluating these outcomes enhances their relevance to clinical conditions, including the common cold and flu. Our study found that the activity of different alkylamides against viruses and immune modulation varies considerably. As summarized in Table 1, alkylamides A, B, and C (Dodeca-2*E*,4*E*,8*Z*,10(*E*/*Z*)-TE acid isobutylamide, Undeca-2*E*-ENE-8,10-Diynoic acid isobutylamide, and Dodeca-2*E*-ENE-8,10-Diynoic acid isobutylamide, respectively) had antiviral activity against the rhinovirus but were ineffective against the influenza virus and may actually be pro-inflammatory, leading to increases in IL-8 secretion. Since the production of IL-8 during a common cold or flu infection leads to increased symptoms, it is proposed that, if these alkylamides were used for the treatment of these infections, an increase in symptoms could be observed [23,24]. Alkylamide D (Dodeca-2*E*,4*E*-Dienoic acid isobutylamide), however, demonstrated potent antiviral activity against both the rhinovirus and influenza virus and led to a decrease in IL-8 synthesis, which, concomitantly, has the potential to lessen symptom duration and severity in patients infected with either of these viruses. This is a desirable effect similar to that observed with the crude *Echinacea* extract. It would be proposed that treatment with this alkylamide during a common cold or flu infection could lead to reduced symptoms associated with the infection. In addition, the selectivity indices for both the 70% *E. purpurea* root extract and alkylamide D (Dodeca-2*E*,4*E*-Dienoic acid isobutylamide) were very high, suggesting their low potential for toxicity.

Clinical studies on the bioavailability and pharmacokinetics of alkylamides in *Echinacea* have focused exclusively on dodeca-2*E*,4*E*,8*Z*,10(*E*/*Z*)-tetraenoic acid isobutylamides (eight/nine or alkylamide A in our study). According to a summary of pharmacokinetic studies of *Echinacea* preparations by Woelkart and Bauer (2007), plasma levels of 10–45 ng/mL of dodeca-2*E*,4*E*,8*Z*,10(*E*/*Z*)-tetraenoic acid isobutylamides have been achieved in an average of 30 min after a single oral dose of tablets, mother tincture, or concentrated ethanolic extracts containing a range of 0.07 mg to 4.3 mg isobutylamides [49]. Concentrated ethanolic extracts demonstrated rapid absorption and exhibited the highest Cmax values per unit dose compared to tablets or mother tinctures [49]. This could be due to the improved solubility and extraction efficiency of alkylamides in ethanol. While these studies confirm that dodeca-2*E*,4*E*,8*Z*,10(*E*/*Z*)-tetraenoic acid isobutylamides are bioavailable, comprehensive clinical trials determining the doses required for the therapeutic efficacy of various alkylamides are lacking. In addition, alkylamide D demonstrated greater efficacy in our study against the rhinovirus and the influenza virus compared to alkylamide A, as well as greater IL-8 suppression, at similar doses. This suggests that alkamide D could achieve therapeutic plasma concentrations at lower doses, but more research is needed to determine the bioavailability and pharmacokinetics of alkylamide D. Synergistic effects between alkylamides and other compounds in the ethanolic extract also remain unexplored. Studies combining pharmacokinetics with treatment outcomes are necessary to determine the effective dose of alkamide D alone and in combination with other alkylamides.

In our study, IL-8 expression was evaluated in THP-1 cells (human monocytes) and three of the four alkylamides, namely A, B, and C (Dodeca-2*E*,4*E*,8*Z*,10(*E*/*Z*)-TE acid isobutylamide, Undeca-2*E*-ENE-8,10-Diynoic acid isobutylamide, and Dodeca-2*E*-ENE-8,10-Diynoic acid isobutylamide, respectively), were found to be immunostimulatory, leading to an increase in IL-8 expression, whereas alkylamide D (Dodeca-2*E*,4*E*-Dienoic acid isobutylamide) was a significant inhibitor of IL-8 secretion. As mentioned previously, a study by Raduner et al. (2006) investigated the immunomodulatory effects of alkylamides on CB2 receptor-dependent and -independent cell lines, as well as human whole blood, and tested three of the four alkylamides tested in our study (A, B, and D) [69]. Their study found that, in human whole blood, these three alkylamides stimulated IL-8 expression (150–225%) at low nanomolar concentrations, whereas in CB2-negative HL60 cells, IL-8 was inhibited or not influenced by any of the alkylamides. And in CB2-positive HL60 cells, alkyalmide D stimulated IL-8 expression, suggesting a CB2-independent response. Cannabinoids have been credited with IL-8 modulation in previous studies [81,82], so it is possible that the cell type and presence or absence of CB receptors play a role in alkylamide’s immunomodulatory activity. Alkylamide D (Dodeca-2*E*,4*E*-Dienoic acid isobutylamide), specifically, as well as alkylamide A, have been shown to bind to CB2 receptors more tightly than endogenous cannabinoids, suggesting there may be important cannabinomimentic effects [68]. In addition, our study employed different cell lines, so one might expect to see different results. Further research would be helpful to confirm the role of Dodeca-2*E*,4*E*-Dienoic acid isobutylamide on IL-8 expression in vivo.

In terms of alkylamide extraction into ethanolic products, a pertinent consideration is plant material sourcing. The alkylamide analysis findings of Mudge et al. differed from those of Bauer and Reminger (1989) and Spelman et al. (2009), with certain alkamides such as Dodeca-2*E*,4*E*-Dienoic acid isobutylamide only occurring in *E. angustifolia* roots in Bauer and Remiger’s original analysis in 1989, vs. occurring in *E. purpurea* roots in Spelman 2009 and Mudge et al.’s analyses (2011) [77,78,80]. These discrepancies point to the natural variation present in different plant source materials, plant parts, and species and are some things to be aware of when sourcing and analyzing plant material for commercial or scientific purposes.

There is also some concern about the stability of the alkylamides in both plant material and extracts, as well as ongoing debate about whether fresh or dried plant material extracts have higher alkylamide content. Herbalists have long advocated for the use of fresh plant material, based on *Echinacea*’s historical use and theories about compounds being lost in the drying or storage process, particularly volatiles and water-soluble polysaccharides. However, the science does not support this [59,75,80,83]. A handful of clinical studies using both dried and fresh extracts of the roots, for example, have shown efficacy in preventing RTI’s and RTI-related complications [35,37,39,42,43,53], and at least three studies quantifying levels of alkylamides, specifically, in both fresh and dried roots indicated no loss of alkylamide content during the drying process [59,75,80]. In fact, one study by Spelman et al. (2009) on *E. purpurea* roots, compared three extraction methods and ratios all in 70% ethanol (fresh 1:2, dry 1:5, and dry 1:11) and found similar yields of alkylamides in all three extracts (slightly higher in the more concentrated dry 1:5 extract) [80]. Similarly, Sun et al. performed tests on supercritical extracts of fresh and dried (both ground and unground) *E. angustifolia* roots and discovered the ground and dried roots yielded the highest alkylamide content of all three groups (*p*< or =0.05) [59]. A few studies have shown that the highly unsaturated alkylamide compounds in *Echinacea* are sensitive to heat and oxidation [84,85], but a study by Liu and Murphy (2007) showed that phenolic-rich extracts of *E. purpurea* roots could prevent oxidation of the alkylamides, as the phenolics are often potent antioxidants [86]. Future studies evaluating the stability of individual alkylamides or standardized *E. purpurea* extracts would be beneficial.

Future research on alkylamides and *Echinacea* extracts should explore the bioavailability and pharmacokinetics of various alkylamides, including Dodeca-2*E*,4*E*-Dienoic acid isobutylamide, associating specific doses with treatment outcomes. The in vitro format of this study may not fully reflect the bioactivity and therapeutic potential of alkylamides in humans, as attainable plasma concentrations could be lower than those used in previous experiments or those needed to achieve therapeutic effects. Testing compound synergism, activity against other pathogens and inflammatory mediators, and exploring complex biological interactions would also be beneficial and would improve the broader applicability of these findings.

## 4. Materials and Methods

For botanical extract preparation, freshly harvested *Echinacea purpurea* roots were received from GAIA Herbs (Brevard, NC, USA) along with a certificate of analysis. All plant material was subsequently verified by qualified botanical specialists using reference keys. A voucher specimen of all plant material was deposited in a repository at the Sonoran University of Health Sciences in Tempe, Arizona. The plants were manually cleaned on the same day received. The plant material was dried at room temperature for 5 days and then ground to a fine powder in a VitaMix blender (Olmsted Township, OH, USA). The plant material was extracted for 24 h at room temperature with constant mixing in 70% ethanol (1:10 weight–volume). The plant–liquid mixture was centrifuged at 3000× *g* for 10 min to remove plant debris, and the supernatant was filtered through a 0.2 µM syringe filter. The final extract was stored at 4 °C in a sterile container. A sample of the extract was dried and was found to contain a nonvolatile solute concentration of approximately 50 mg/mL extract.

For alkylamides preparation, the following alkylamides present in *Echinacea purpurea* roots, as previously identified [77,78,80], were obtained from Chromadex: Dodeca-2*E*,4*E*,8*Z*,10*E*(*Z*)-tetraenoic acid isobutylamide, undeca-2*E*-ENE-8,10-diynoic acid isobutylamide, dodeca-2*E*-ENE-8,10-diynoic acid isobutylamide, and dodeca-2*E*,4*E*-dienoic acid isobutylamide. Stock solutions of each alkylamide were prepared in DMSO at a concentration of 1 mg/mL and stored at −20 °C.

For caffeic acids preparation, the following caffeic acids present in *Echinacea purpurea* roots, as previously identified [83,87], were obtained from ChromaDex (Irvine, CA, USA): echinacoside, cafteric acid, chlorogenic acid, cichoric acid, and dicaffeoylquiunic acid. Stock solutions of each caffeic acid were prepared in DMSO at a concentration of 1 mg/mL and stored at −20 °C.

For cell lines and viruses, for the influenza virus, MDCK cells (ATCC. Madin–Darby Canine Kidney (MDCK) Cells (ATCC CCL-34). Madison, WI, USA) were maintained with Dulbecco’s Minimal Essential Media (DMEM, Corning) (Corning, NY, USA)) supplemented with 10% heat-inactivated fetal bovine serum and 1% antibiotic–antimycotic. The MDCK cells were maintained at 37 °C, with 5% CO_2_ in a humidified chamber. For the rhinovirus, HeLaH1 cells (ATCC CRL-1958) were maintained in Dulbecco’s Modified-Minimal Essential Medium (DMEM, Corning) supplemented with 10% heat-inactivated fetal bovine serum and 1% antibiotic–antimycotic. The HeLaH1 cells were maintained at 37 °C, with 5% CO_2_ in a humidified chamber. Human rhinovirus 16, designated as VR-283, was provided by ATCC. Human influenza virus H1N1 (A/USSR/90/77 (ATCC VR-1894), United States, 1934) was provided by ATCC.

For the rhinovirus-16 (VR-283) plaque assay, the HeLaH1 cells were infected with 100–200 pfu (plaque forming units) of rhinovirus in the presence of varying concentrations of *Echinacea purpurea* root 70% EtOH extract or alkylamides for 1 h at 37 °C followed by incubation in media containing *Echinacea* extract or alkylamides for 3 days at 33 °C. No hard medium overlay was used in this assay. The plaques were visualized by staining with 0.1% crystal violet in 20% ethanol. Solvent controls (70% ethanol for the ethanolic extract and DMSO for individual compounds) were prepared at the same volumes corresponding to the treatment doses and included in the assay. These were tested to ensure that they had no significant effect on plaque formation compared to the untreated controls.

For the influenza virus H1N1 (VR-1894) plaque assay, human influenza virus H1N1 (ATCC VR-1894) was propagated in MDCK cells. For the viral assays, MDCK cells were washed twice with PBS. The virus was diluted in DMEM with 0.35% BSA. Cells were infected with 100–200 pfu (plaque-forming units) of diluted influenza virus in the presence of varying concentrations of *Echinacea purpurea* root EtOH extract or alkylamides for 1 h at 37 °C. The cell monolayer was washed twice with PBS followed by an overlay containing DMEM, 0.02% DEAE-Dextrose, 0.03% MgSO_4_, 1 µg/mL TPCK-Trypsin, 0.6% agarose, and comparable amounts of the *Echinacea* extract or alklyamides. The cells were incubated for 3 days at 37 °C. Plaques were visualized by staining with 0.1% crystal violet in 20% ethanol. Solvent controls (70% ethanol for the ethanolic extract and DMSO for individual compounds) were prepared at the same volumes corresponding to the treatment doses and included in the assay. These were tested to ensure they had no significant effect on plaque formation compared to the untreated controls.

For the cytokine expression assay, THP-1 cells (ATCC TIB-202) were maintained in RPMI media (Corning) supplemented with 10% heat-inactivated fetal bovine serum and 1% antibiotic–antimycotic. THP-1 cells were maintained at 37 °C, with 5% CO_2_ in a humidified chamber. Prior to treatment (24 h), the cells were matured by treatment with 10 µM PMA. Differentiated THP-1 cells were left untreated, treated with *Echinacea* extracts (50 µg/mL), or treated with alkylamides (1 µg/mL) followed by +/− treatment with lipopolysaccharide (LPS) (1 µg/mL to induce cell activation and cytokine production) for 24 h. The cell culture supernatant was collected at 24 h and analyzed by ELISA (Boster (Pleasanton, CA, USA)) for IL-8. The expression level of IL-8 (pg/mL) was measured via absorbance at 450nm using a microplate reader and compared to a standard curve. Mock controls (media ± 1 µg/mL LPS) were included to establish the baseline IL-8. Vehicle controls (70% ethanol for the ethanolic extract and DMSO for alkylamides) were prepared at the same volumes corresponding to the treatment doses. Solvent controls were tested both with and without LPS to confirm their lack of impact on IL-8 production

For the LC-MS analysis of alkylamides in *E. pupurea* extract, an LC-MS analysis of alkylamides was performed on a UHPLC system (Agilent 1290 Infinity II) equipped with binary pumps, a diode array detector, coupled to a single quadrupole mass spectrometer (Agilent LC/MSD XT). Chromatographic separation was carried out on a C18 column (Zorbax SB C18 RRHD, 150 mm × 2.1 mm i.d., 1.8 μm, Agilent (Santa Clara, CA, USA)) at room temperature. The mobile phases consisted of 0.1% (*v*/*v*) formic acid in water (A) and acetonitrile (B) with the elution gradient set as 10–100% B in 40 min. The flow rate was 0.4 mL/min. The MS data were collected in dual polarity scan mode with capillary voltages of 4000 V and a nebulizer pressure of 35 psi. The gas temperature was set at 350 °C with a gas flow of 12 L/min. The quadrupole temperature was set at 100 °C. Undec-2-Ene-8,10-Diynoic acid isobutylamide, Dodec-2*E*-Ene-8,10-Diynoic acid isobutylamide, and Dodeca-2*E*,4*E*-Dienoic acid isobutylamide were dissolved in 30:70 H_2_O:MeOH at 1000 µg/mL. Dodeca-2*E*,4*E*,8*Z*,10*Z*-Tetraenoic acid isobutylamide was dissolved in methanol at 250 µg/mL. Calibration standards were generated from the stock at concentrations of 0.5, 1, 5, 25, 50, 125, and 250 µg/mL. Calibration and quantification of alkylamides were conducted at 330 nm.

For the cytotoxicity assay/CC_50_, to determine the cytotoxicity of the *E. purpurea* extracts and alkylamides, either HeLaH1 or MDCK cells were seeded in a clear, flat-bottomed 96-well plate at a cell density of 1 × 10^4^ cells/well in a final volume of 200 µL of media and incubated for 24 h at 37 °C with 5% CO_2_. The cells were dosed with increasing concentrations (10–1000 µg/mL) of either *E. purpurea* extract or individual alkylamides and were incubated for another 24 h under the same conditions. The plates were washed with 200 µL of media three times to remove any botanical residue and brought to a final volume of 200 µL of media/well. Added to each well were 20 µL of MTS reagent (Abcam (Cambridge, England, UK), and the plates were incubated for 2 h at 37 °C with 5% CO_2_. The absorbance was measured at 490 nm using a microplate reader. The CC_50_ (dose to reduce cell viability by 50%) was determined by plotting a concentration vs. cell viability dose–response curve and analyzing viability as a percentage of the control.

For the statistical analysis, to determine the statistical significance between the sample data collected and the null hypotheses, statistical analyses were conducted using GraphPad’s *t*-test calculator. One sample *t*-tests were conducted to analyze the sample test results compared to the respective controls, and *p*-values were calculated to determine the statistical significance of the observed differences. The following *p*-values were categorized and interpreted as statistically significant:

- 0.01 ≤ *p* < 0.05: Significant (*p* < 0.05);
- 0.001 ≤ *p* < 0.01: Highly significant (*p* < 0.01);
- *p* < 0.001: Extremely significant (*p* < 0.001).

For the selectivity index (SI) calculation, the selectivity index (SI) was calculated using the following formula: SI = CC_50_/IC_50_. CC_50_ represents the treatment dose that caused 50% toxicity in host cells, and IC_50_ represents the dose that inhibited 50% of viral plaque formation (toxic dose/effective dose).

## 5. Conclusions

Although previous research suggests that the alkylamides present in *Echinacea* may be responsible for reducing the symptoms associated with the common cold or flu, the role of which specific alkylamides and their target (i.e., immune and/or antiviral) have not been well elucidated or established. This study tested the antiviral and cytokine regulatory activity of an *E. purpurea* root ethanol extract and various pure alkylamides and found that one specific alkylamide, Dodeca-2*E*,4*E*-Dienoic acid isobutylamide, had potent antiviral activity against the rhinovirus (the causative agent of most common colds) and the influenza virus and potent inhibition of IL-8 cytokine production, which is responsible for many symptoms associated with these viral infections. The broad activity and low cytotoxicity of this specific alkylamide support its potential use for the treatment of rhinovirus and influenza virus infections.

## Figures and Tables

**Table 1:** Summary of activities of various alkylamides. Alkylamides A–D (Dodeca-2*E*,4*E*,8*Z*,10(*E*/*Z*)-TE acid isobutylamide, Undeca-2*E*-ENE-8,10-Diynoic acid isobutylamide, Dodeca-2*E*-ENE-8,10-Diynoic acid isobutylamide, and Dodeca-2*E*,4*E*-Dienoic acid isobutylamide, respectively) were compared to evaluate their comprehensive antiviral and immunomodulatory properties. The **+** values indicate relative levels of inhibitory activity (arbitrary values), and the **−** values indicate no inhibitory activity.

Sample	Alkylamide	Anti-Rhinovirus Activity	Anti-Influenza Virus Activity	Inhibition of IL-8 Secretion
Alk A	Dodeca-2(*E*), 4(*E*), 8(*Z*), 10(*E*/*Z*)-TE acid isobutylamide	**+++**	**−**	**−**
Alk B	Undeca-2*E*-ENE-8,10-Diynoic acid isobutylamide	**++**	**+**	**−**
Alk C	Dodeca-2*E*-ENE-8,10-Diynoic acid isobutylamide	**++++**	**−**	**−**
Alk D	Dodeca-2(*E*),4(*E*)-Dienoic acid isobutylamide	**++++**	**++**	**+**

## Data Availability

All data analyzed during this study are included in this published article. No additional data sets were used.
